# Prevalence of monoclonal gammopathy of undetermined significance (MGUS) using a sensitive mass spectrometry assay in young individuals 10–49 years of age: a population-based study from the National Health and Nutritional Examination Survey

**DOI:** 10.1038/s41408-026-01518-7

**Published:** 2026-05-25

**Authors:** Shaji Kumar, David Murray, Dirk R. Larson, Ola Landgren, P. Leif Bergsagel, James R. Cerhan, S. Vincent Rajkumar

**Affiliations:** 1 Division of Hematology, Department of Medicine, Mayo Clinic, Rochester, MN USA; 2 Department of Laboratory Medicine and Pathology, College of Medicine, Mayo Clinic, Rochester, MN USA; 3 Division of Biostatistics, Department of Medicine, College of Medicine, Mayo Clinic, Rochester, MN USA; 4 Sylvester Myeloma Institute, Sylvester Comprehensive Cancer Center, University of Miami, Miami, FL USA; 5 Division of Hematology and Oncology, Department of Medicine, Mayo Clinic, Scottsdale, AZ USA; 6 Division of Epidemiology, Department of Health Sciences Research, Mayo Clinic, Rochester, MN USA

**Keywords:** Epidemiology, Oncogenesis

## Abstract

The prevalence of monoclonal gammopathy of undetermined significance (MGUS) increases with age, but the study of age of onset and the prevalence of MGUS in younger individuals has been limited by lack of sensitive serum protein testing methods. In addition, there is marked racial disparity in the prevalence in Black individuals compared with White individuals and it is important to determine if MGUS has an earlier age of onset in Black individuals. In this first large mass spectrometry-based population-based screening study of MGUS, we evaluated 12,378 individuals (3598 White, 4075 Black, 4147 Mexican Americans, and 558 others) aged 10–49 years of age. The study used serum samples from the National Health and Nutritional Examination Survey (NHANES) III. MGUS was identified in 177 persons (1.28%, 95% CI 0.95, 1.60). MGUS was detectable in 0.2% of individuals age 10–19 years and the prevalence increased with age to 0.88%, 1.46%, and 2.82% in individuals ages 20–29, 30–39, and 40–49, respectively. The age-adjusted prevalence of MGUS was significantly higher in Black individuals compared with White individuals, 1.49% (1.13, 1.95) versus 0.82% (0.57, 1.18), *P* = < 0.01. MGUS had an earlier age of onset in Black individuals; the increased prevalence of MGUS among Black compared with White individuals was apparent at age 30–39 years, 2.94% vs 1.42%, and this disparity increased further in the age group 40–49 years to 5.2% vs 2.8%, respectively. There was no significant difference in the prevalence of MGUS between White individuals and Mexican American individuals in the overall cohort, or within any specific age group. The study using a highly sensitive mass spectrometry-based assay demonstrates onset of monoclonal gammopathy in the second decade of life, with a higher prevalence among the Black individuals with comparable prevalence among the White and Mexican individuals, a disparity that increases with advancing age.

## Introduction

Monoclonal gammopathy of undetermined significance (MGUS) is a common premalignant plasma cell neoplasm present in approximately 3–5% of the general population above the age of 50 [1, 2]. MGUS is diagnosed based on the presence of abnormal monoclonal (M) protein (intact immunoglobulins or free light chains secreted by the clonal plasma cells) in the serum or urine of normal asymptomatic individuals. MGUS is of major clinical significance since it is the precursor for malignancies such as multiple myeloma (MM), Waldenstrom macroglobulinemia (WM), and solitary plasmacytoma [3–7]. It is also causally associated with a wide variety of non-malignant disorders including immunoglobulin light chain (AL) amyloidosis, cryoglobulinemia, proliferative glomerulonephritis, and light chain deposition disease, collectively referred to as monoclonal gammopathies of clinical significance (MGCS). Epidemiological studies suggest that MGUS is present many years prior to diagnosis, and that the prevalence of MGUS varies significantly by race, age, and gender [8]. However, existing data is limited by low sensitivity of testing methods utilized with no good studies to accurately estimate the age of onset of MGUS and to discriminate the extent of disparities across various groups. This information is needed to develop targeted screening strategies in high risk populations [9, 10]. In addition, with the etiology of monoclonal gammopathies still unclear, a better understanding of the prevalence, age of onset, and potential risk factors can shed light on the biology of this condition [11, 12].

Initial epidemiological studies from the Olmsted County suggested the prevalence to be ~3% among those over 50, however this study included almost exclusively white individuals [10]. Subsequently we studied a cohort more representative of the US population using samples from the National Health and Nutritional Examination Survey (NHANES), demonstrating a prevalence of 2.4% but with significant racial disparity, with a 2-to-3-fold higher prevalence of MGUS in Black individuals compared to White individuals [2, 13]. This racial disparity is felt to be the primary reason driving the higher prevalence of myeloma in Black individuals. We have also demonstrated a higher prevalence of MGUS among first degree relatives of individuals with monoclonal plasma cell disorders [6]. There are limited data on the age of onset of MGUS, its prevalence in individuals younger than 50 and if the higher prevalence of MGUS in Black individuals is related to an earlier age of onset. We have previously reported on the prevalence of MGUS in persons 10–49 years of age using samples from NHANES, but although we found a higher prevalence of MGUS among Black individuals in the 40–49 years age group, the lack of a sensitive test to detect small M proteins greatly limited our investigations, and that of others, in estimating the true age of onset and the prevalence of MGUS by age, race, and gender in these younger age groups [2]. More recently, highly sensitive mass spectrometry based testing of monoclonal proteins has become available, improving our ability to detect monoclonal proteins compared to traditional electrophoretic methods and immunofixation [14–16].

In this paper we report on the first population-based study to determine the prevalence of MGUS using sensitive mass spectrometry methods in younger individuals aged 10–49 years using the same cohort from NHANES that we have previously studied using standard electrophoretic and immunofixation methods [2]. The goals of our study were to provide precise estimates of the prevalence of MGUS in the overall population, determine the age of onset of MGUS, and to study differences in prevalence based on age, gender, and race.

## Methods

### Study population and serum samples

The details of the cohort studied have been previously described. Briefly, the data for this paper comes from NHANES III (1988–1994), a large population-based cross-sectional study that includes in-person home interviews and medical examinations conducted by the Centers for Disease Control and Prevention (CDC) to assess the health and nutritional status of adults and children in the United States [13, 17, 18]. Participant identification is based on a stratified multistage complex sample design to select a nationally representative sample of the civilian, non-institutionalized US population, with oversampling (higher sampling rates) of older adults and non-Hispanic Blacks and Mexican Americans, and other groups. There were 14,153 individuals in the NHANES III sample who were 10–49 years old at home interview who participated in the medical examination component. Of these individuals, 12,378 individuals (87%) had serum samples available for MGUS testing. Because of the loss of observations due to insufficient serum samples used for the MGUS testing, the sample weights provided by NCHS for the MEC sample were further adjusted by post-stratification using age (10–11, 12–19, 20–29, 30–39, 40–49) by sex by race/ethnicity (non-Hispanic white, non-Hispanic black, Mexican American, and other races) categories to make the weighted sample for individuals in NHANES with MGUS evaluation representative of the US population. These categories were similar to those used by NCHS for post-stratifying the original MEC weights. Survey data, including demographics, health status, health disorders, behaviors and so on, were collected through household interviews. Physical examinations and blood collection were conducted at a mobile examination center.

### Mass spectrometry and electrophoresis testing for monoclonal proteins

Mass spectrometry was performed on all samples from NHANES with adequate volume available for testing at the Protein Immunology Laboratory, Mayo Clinic, Rochester, MN, USA, using laboratory techniques that have been well described previously [19, 20]. Briefly, immunopurification was achieved through incubation with CaptureSelect resin and subsequent elution and reduction of the adsorbed immunoglobulins using 30 μL of 20 mM TCEP + 00.1% TFA. Sample eluent (0.5 μL, purified patient Igs in 0.1% TFA containing 10 mM TCEP) was combined with CHCA matrix (0.7 μL) and spotted onto a 96-well microScout polished steel Bruker target (Bruker Daltonics) using a single application spotting method. Analysis was performed in positive ion mode with summation of 500 laser shots using a MALDI-TOF mass spectrometer (Bruker Microflex LT, Germany). In-house designed software is used for spectra analysis. The software automatically imports individual patient spectra from each immunopurification and smoothes them via the Savitzky-Golay method. Spectra are displayed in three graphs, each lg subtype (G, A and M) overlaid with the lambda and kappa spectra. Standard electrophoresis and confirmation with serum immunofixation for comparison was previously done on almost all of these samples as reported earlier [10]. For standard electrophoresis testing, conventional agarose-gel electrophoresis was performed on available sera, and samples with an equivocal or definite M-protein present on electrophoresis were subjected to immunofixation and serum-free light-chain assay. All testing and interpretation were done by individuals blinded to demographic and other details pertaining to the samples.

### Prevalence estimates and risk factors

Once testing was completed, data were transferred to the National Center for Health Statistics to link to the NHANES Public Use files that contain the serologic and demographic data needed for these analyses. Once the data were released on the NHANES web site, we used the publicly available data for this study. The prevalence of MGUS was estimated in the total population, as well as for Whites, Blacks, and Mexican Americans separately, and by age, gender and other known risk factors. Individuals with a monoclonal protein detected by either electrophoresis and immunofixation or through mass spectrometry were considered to have MGUS. Those with indeterminate results or inadequate samples were all considered as negative.

### Statistical analysis

Demographics and baseline data are reported using standard descriptive statistics. All analyses accounted for the complex multistage probability cluster sampling design of the NHANES surveys, including clustering, stratification, and weighting. Furthermore, due to the exclusion of the records of those with insufficient serum samples for MGUS testing, the sample weights provided by NCHS were further adjusted by post-stratification using age (10–11, 12–19, 20–29, 30–39, 40–49) by sex by race/ethnicity (non-Hispanic white, non-Hispanic black, Mexican American, and other races) categories to make the weighted study sample representative of the US population. Prevalence rates were calculated by dividing the sample weighted number of persons with MGUS by the sample weighted number of total persons (with or without MGUS). Logistic regression was used to perform adjusted analyses for potential confounders including age, race, and sex [21]. The relationship of age and MGUS prevalence was examined using a restricted cubic spline in logistic regression [21, 22]. Risk factors for MGUS were evaluated utilizing survey information available in the NHANES III data. Specifically, we analyzed potential risk factors that have been reported in previous studies as having potential associations with MGUS—namely, age, race/ethnicity, obesity, and socioeconomic status (SES) [10, 12, 13, 23–25]. The poverty index ratio was used as a proxy marker for SES. All analyses were conducted using SAS version 9.4M8 (SAS Institute Inc., Cary, NC, USA) and R version 4.4.1 (R Foundation for Statistical Computing, Vienna, Austria). All statistical tests were two-sided and *p*-values less than 0.05 were considered statistically significant. Because of the small number of individuals with MGUS in the NHANES III sample, confidence intervals and *p*-values should be interpreted with caution.

## Results

### Prevalence

**Fig. 1:**
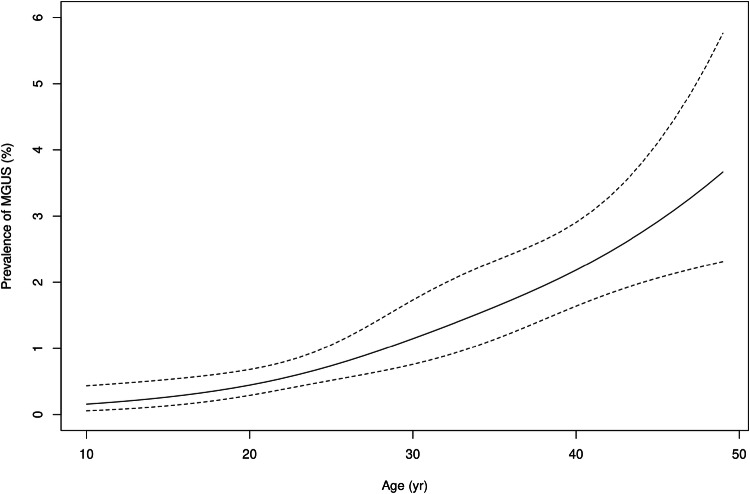
Prevalence of MGUS among 10–49-year-olds in NHANES III, showing increasing prevalence by age. The solid line is the estimated prevalence from 3-knot cubic restricted regression spline using logistic regression unadjusted for any covariates. The dash lines show the upper and lower 95% pointwise confidence limits.

**Fig. 2:**
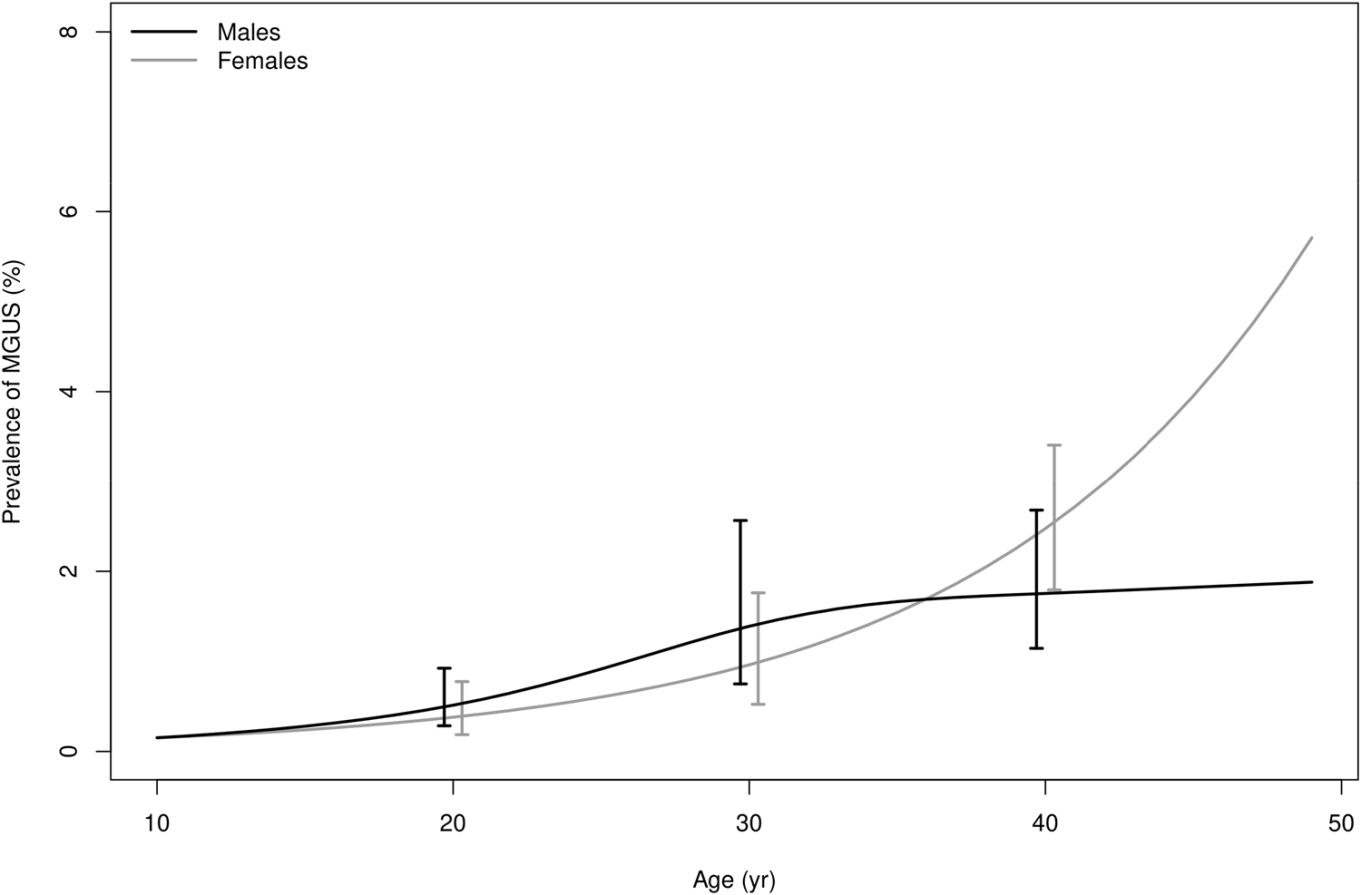
Prevalence of MGUS among 10–49-year-olds in NHANES III, showing increasing prevalence by age among both sexes. The solid line is the estimated prevalence from 3-knot cubic restricted regression spline using logistic regression unadjusted for any covariates. The dash lines show the upper and lower 95% pointwise confidence limits.

Of 14,153 study subjects aged 10 to 49 years of age enrolled in NHANES III, stored serum samples to test for monoclonal proteins were available for testing in 12,378 persons (4075 non-Hispanic Black individuals [considered ‘Black individuals’]; 4147 Mexican Americans; 3598 non-Hispanic whites [considered ‘white’]; and 558 ‘others’). The demographics of the overall population, and by race and by presence or absence of MGUS are as shown in supplementary table 1. We pooled mass spectrometry results with prior electrophoresis and immunofixation results to determine the overall prevalence of MGUS. MGUS was detected in 177 persons (1.28%, 95% CI 0.95, 1.60); the prevalence by age, gender, and race are shown in Table 1 and supplementary Table 2. MGUS was detectable in 0.2% of individuals age 10–19 years. The median age of the 177 patients with MGUS was 38 years compared with 27 years for those with a negative test (*n* = 12,201). Patients with MGUS included 51 (28.8%) Whites, 80 (45.2%) Blacks, 42 (23.7%) Mexican Americans and 4 (2.3%) Other. The distribution of the 177 patients with MGUS, by age, gender and race are shown in Table 2. The overall prevalence of MGUS increased with age; 0.20% (95% CI; 0.02, 0.37) among persons 10–19 years to 2.82% (95% CI; 1.90, 3.74) among those 40–49 years (Fig. 1), reflecting a significant increase across each age group (Table 3). MGUS was detected in 100 of 6666 sampled women (1.44%, 95% CI; 0.99, 1.89), as compared with 77 of 5712 sampled men (1.11%, 95% CI; 0.73, 1.49), *p* = 0.26. The increase in prevalence with age was observed in both men and women (Fig. 2).

**Table 1:** Prevalence of MGUS (%) by race, age, and gender.

Variable (# with MGUS)	Number with MGUS	Blacks (80)^a^ % (95% CI)	Whites (51)^a^ % (95% CI)	Mexican American (42)^a^ % (95% CI)	Total (177)^b^ % (95% CI)
**Age group, years**					
10–19	14	0.37 (0.01, 0.72)	0.17 (0.00, 0.36)	0.38 (0.00, 0.75)	0.20 (0.02, 0.37)
20–29	28	0.70 (0.13, 1.27)	0.77 (0.27, 1.27)	0.56 (0.12, 1.01)	0.88 (0.43, 1.34)
30–39	53	2.94 (1.89, 3.99)	1.42 (0.54, 2.31)	0.83 (0.20, 1.47)	1.46 (0.78, 2.13)
40–49	82	5.23 (3.30, 7.16)	2.75 (1.59, 3.92)	2.59 (1.36, 3.83)	2.82 (1.90, 3.74)
**Sex**					
Male	77	1.99 (1.30, 2.67)	1.04 (0.55, 1.53)	0.98 (0.55, 1.41)	1.11 (0.73, 1.49)
Female	100	2.06 (1.41, 2.72)	1.49 (0.86, 2.12)	0.75 (0.39, 1.11)	1.44 (0.99, 1.89)
**Total**	177	2.03 (1.53, 2.52)	1.26 (0.82, 1.71)	0.87 (0.57, 1.18)	1.28 (0.95, 1.60)

*CI* confidence interval.

^a^Number in parentheses indicates number of people with MGUS in that race/ethnicity group.

^b^Includes ‘Other’ race/ethnicity group.

**Table 2:** Characteristics of 177 individuals with MGUS (%) by race/ethnicity.

Total, n	Black (*N* = 80)	White (*N* = 51)	Mexican American (42)
**Age group, years, % (95% CI)**			
10–19	5.27 (2.05, 8.48)	3.29 (0.00, 7.78)	13.2 (0.00, 28.4)
20–29	9.03 (4.98, 13.1)	14.8 (5.85, 23.8)	20.4 (7.19, 33.6)
30–39	38.4 (30.0, 46.9)	31.6 (18.3, 44.8)	22.2 (3.94, 40.5)
40–49	47.3 (39.1, 55.5)	50.4 (42.3, 58.4)	44.2 (25.2, 63.3)
**Sex, % (95% CI)**			
Male	45.8 (35.8, 55.9)	41.6 (30.5, 52.8)	58.9 (44.2, 73.7)
Female	54.2 (44.2, 64.2)	58.4 (47.2, 69.5)	41.1 (26.3, 55.8)

*CI* confidence interval.

**Table 3:** Prevalence of MGUS by risk factor.

Variable	Level	Unadjusted		Adjusted^a^	
		Prevalence	*p*-value	Prevalence	*p*-value
Age group, years	10–19	0.20 (0.02, 0.37)	10–19 vs 20–29: 0.008	0.19 (0.07, 0.45)	10–19 vs 20–29: 0.011
	20–29	0.88 (0.43, 1.34)	10–19 vs 30–39: <0.001	0.85 (0.48, 1.47)	10–19 vs 30–39: <0.001
	30–39	1.46 (0.78, 2.13)	10–19 vs 40–49: <0.001	1.39 (0.86, 2.26)	10–19 vs 40–49: <0.001
	40–49	2.82 (1.90, 3.74)	20–29 vs 30–39: 0.147	2.71 (1.99, 3.68)	20–29 vs 30–39: 0.145
			20–29 vs 40–49: <0.001		20–29 vs 40–49: <0.001
			30–39 vs 40–49: 0.008		30–39 vs 40–49: 0.006
Sex	Male	1.11 (0.73, 1.49)		0.74 (0.48, 1.14)	
	Female	1.44 (0.99, 1.89)	0.205	0.93 (0.66, 1.32)	0.264
Race	White	1.26 (0.82, 1.71)	W vs B: 0.020	0.82 (0.57, 1.18)	W vs B: 0.005
	Black	2.03 (1.53, 2.52)	W vs MA: 0.167	1.49 (1.13, 1.95)	W vs MA: 0.560
	Mexican American	0.87 (0.57, 1.18)	B vs MA: <0.001	0.70 (0.49, 1.01)	B vs MA: <0.001
	Other	0.65 (0.17, 2.47)		0.47 (1.11, 2.05)	
Body Mass Index	Normal/overweight	1.16 (0.89, 1.50)		0.82 (0.60, 1.12)	
	Obese	1.89 (1.10, 3.23)	0.146	0.91 (0.46, 1.82)	0.729
Poverty	Below poverty	1.22 (0.84, 1.60)		0.72 (0.47, 1.13)	
	Not below poverty	1.51 (0.34, 2.69)	0.637	1.19 (0.49, 2.90)	0.337
Region	Northeast/Midwest	1.48 (0.98, 2.23)		0.72 (0.51, 1.02)	
	South/West	1.12 (0.84, 1.49)	0.288	0.97 (0.61, 1.55)	0.256

Age group: adjusted for sex and race.

Sex: adjusted for age and race.

Race: adjusted for age and sex.

BMI: adjusted for age, sex, and race.

Poverty: adjusted for age, sex, and race.

Region: adjusted for age, sex, and race.

^a^Adjusted models:

**Fig. 3:**
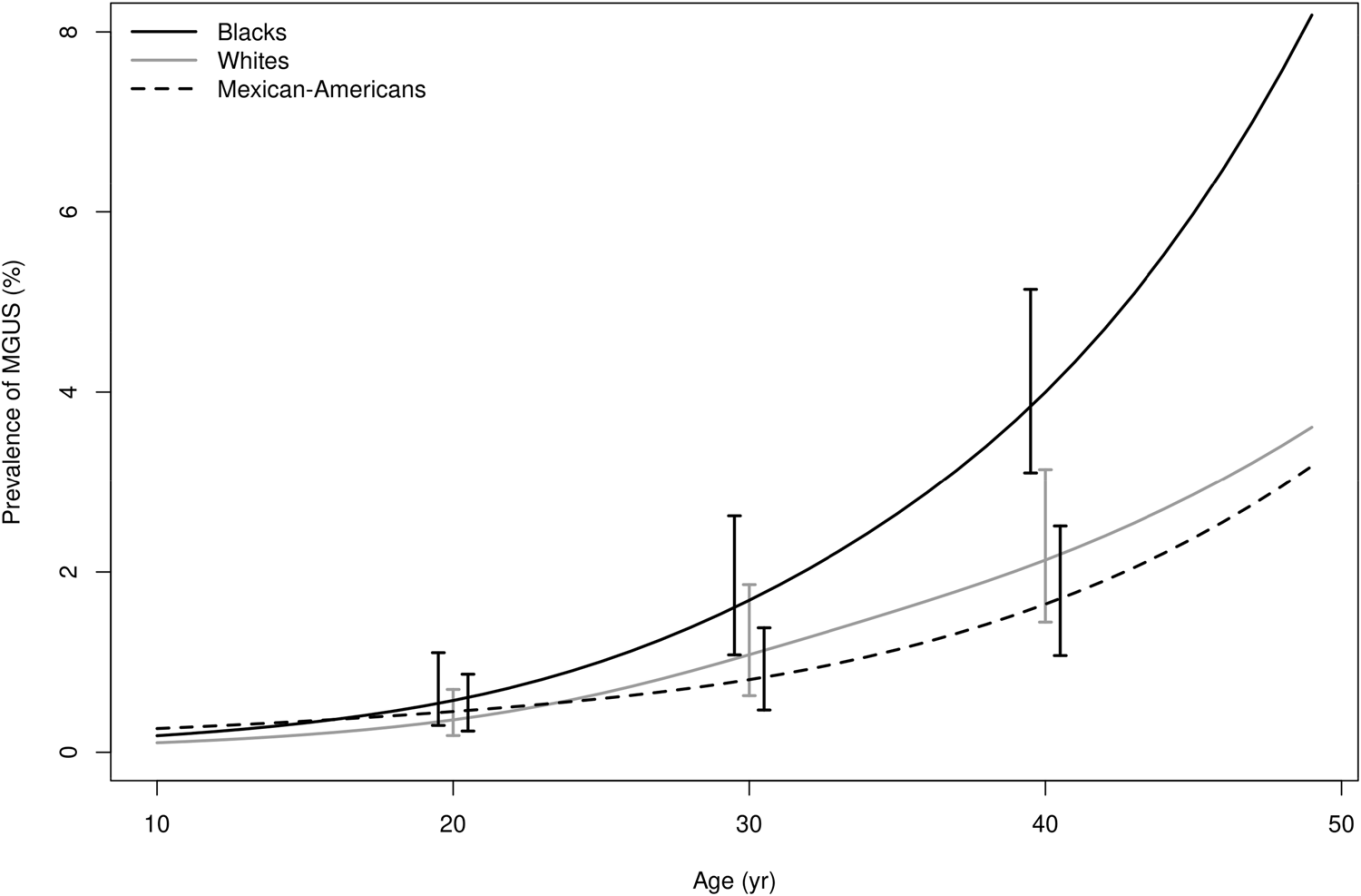
Prevalence of MGUS among 10–49-year-olds in NHANES III showing increasing prevalence by age by selected race-and Hispanic ethnicity. The solid line is the estimated prevalence from 3-knot cubic restricted regression spline using logistic regression unadjusted for any covariates. The dash lines show the upper and lower 95% pointwise confidence limits.

Overall, the prevalence (95% CI) of MGUS by race-ethnicity was 2.03% (1.53, 2.52) for Blacks, 1.26% (0.82, 1.71) for Whites, 0.87% (0.57, 1.18) for Mexican American and 0.65% (0.17, 2.47) for Other. The age and sex adjusted prevalence of MGUS in Black individuals (1.49%, 95% CI; 1.13, 1.95) was higher compared with both White individuals (0.82%, 95% CI; 0.57, 1.18, *p* = 0.005) and Mexican Americans (0.70% 95% CI; 0.49, 1.01; *p* < 0.001) (Table 3). MGUS had an earlier age of onset in Black individuals, with the increased prevalence of MGUS among Black individuals compared with White individuals apparent at age 30–39 years, 2.94% vs 1.42%, and increasing further in the age group 40–49 years to 5.2% vs 2.8%, respectively. (Table 1, Fig. 3). There was no significant difference in the prevalence of MGUS between White individuals and Mexican American individuals in the overall cohort, or within any specific age group. The youngest age at which we detected MGUS was 14, 10, and 10 in White, Black, and Mexican American individuals, respectively. In the age-group 10–19 (predominantly children), there were 14 persons with MGUS, 5 Black, 2 White, and 7 Mexican American.

### Risk factors

Although the number of MGUS cases overall were small, we explored risk factors based on prior studies including: BMI [12, 26–28] and SES (categorized as high versus low by using poverty index ratio <1 versus ≥1) (Table 3 and Supplementary Tables 3) [12, 23–25]. Prevalence of MGUS was not statistically associated with BMI; the age, gender and race adjusted prevalence among obese individuals was 0.91% (95% CI; 0.46, 1.82) compared to non-obese individuals 0.82 (0.60, 1.12), *p* = 0.729. Poverty income-ratio was not significantly associated with the prevalence of MGUS after adjustment for age, gender, and race, *p* = 0.337. There was no association with the region of the country with an adjusted prevalence of 0.72% (95% CI; 0.51, 1.02) for Northeast/Midwest and 0.97% (95% CI; 0.61, 1.55) for South and West, *p* = 0.256.

## Discussion

This study represents the most comprehensive assessment of the prevalence of MGUS in a large population using one of the most sensitive techniques available currently, namely mass spectrometry, to detect presence of a monoclonal protein in the serum. The results presented here are highly impactful as it enhances our current understanding regarding the age of onset of MGUS, and the prevalence of MGUS in persons less than 50 years of age by age-group, gender, and race. The results are robust and derive from a population-based cohort that is a nationally representative sample of the US population, with oversampling of non-Hispanic blacks and Mexican Americans, and other groups allowing for more precise estimation of the prevalence in these groups.

Our study provides several new important findings that are important for our understanding of MGUS biology and for clinical practice. First, we provide definitive evidence of onset of MGUS as early as the second decade of life. Second, we provide accurate data on the prevalence of MGUS in the general population age less than 50 years stratified by age-group, gender, and race using sensitive mass spectrometry. These data are important to our understanding of the epidemiology of MGUS and can also help in clinical practice. They fill an important gap in our knowledge base since most prior studies were conducted in persons over the age of 50 and using standard electrophoretic methods with low sensitivity. Third, we provide clear evidence for the first time that there is a significantly higher risk of MGUS in Black people that starts at a young age-group of 30–39 [5, 8, 13, 29]. Finally, we find that the higher prevalence of MGUS in men compared to women that has been reported in studies conducted in persons age 50 and over is not seen in the age group below 50. There have been earlier studies that have investigated the role of hormonal factors in prevalence of MGUS, and the fact that until menopausal years there is no difference in prevalence by gender that we find in this study is of biologic importance.

Our study builds on the findings of multiple population-based screening studies over the years that have examined the prevalence of MGUS in the general population [10, 15, 30–32]. In one of the earliest studies, screening of individuals residing in Olmsted County, Minnesota using serum protein electrophoresis suggested a prevalence of MGUS of 3.2 percent among persons 50 years of age or older and 5.3 percent among those 70 years of age or older [10]. Similar estimates have been arrived at in other studies as well but had been based on a predominantly white population from the United States and Europe. One of the largest population-based studies is the iSTOPMM study from Iceland that screened over 75,000 individuals over age 40 residing in Iceland [33]. A higher prevalence among blacks was reported initially in a study of 917 individuals from Ghana, where 54 individuals were found to have MGUS, yielding an age-adjusted prevalence of 5.84% [5]. In an initial study using stored samples from 12,482 individuals included in the NHANES III or NHANES 1999–2004 who were 50 years or older, MGUS was identified in 365 participants (2.4%) [13]. Adjusted prevalence of MGUS was significantly higher (*P* < 0.001) in blacks (3.7%) compared with whites (2.3%) (*P* = 0.001) or Hispanics (1.8%). All these studies were restricted to individuals over age 50. We studied 12,372 individuals younger than 50 years enrolled in the NHANES studies and found that, prevalence of MGUS was significantly higher in blacks (0.88%, 95% CI 0.62–1.26) compared with whites (0.22%, 95% CI 0.11–0.45), but our ability to discriminate differences in prevalence in age groups 30–39 and lower, and to determine age of onset of MGUS were limited by the low sensitivity of standard electrophoresis methods. In this regard the key findings of this study provide important information to aid our studies of MGUS pathogenesis, and inform clinical practice with regards to screening, studying disease associations, risk-stratification, and management. The prevalence in the current study, as with prior determinations, is based on a single assessment and it is possible that a few of them may have disappeared over time but based on prior studies we anticipate this to be very few, if any.

The mass spectrometric techniques used in our study are well validated and is more sensitive and specific for diagnosis of MGUS [14, 16, 29, 34]. Increasingly, mass spectrometry is replacing conventional protein electrophoretic methods, for many reasons including efficiency of laboratory workflow in addition to the sensitivity [35]. In an earlier study, we identified 300 individuals diagnosed with MGUS or related gammopathy who had a prior negative work-up for monoclonal proteins as part of the Olmsted County MGUS screening study. Two contemporary highly sensitive tests (matrix-assisted laser desorption/ionization-time of flight (MALDI-TOF) and monoclonal immunoglobulin rapid accurate mass measurements (miRAMM)) along with traditional immunofixation were performed on the Olmsted County baseline and MGUS diagnostics serum samples [15]. We studied 226 patients with available samples using mass spectrometry-based detection methods and identified a monoclonal protein in 24 patients (10.6%) by immunofixation, 113 patients (50%) by MADLI-TOF mass spectrometry, and 149 patients (65.9%) by miRAMM mass spectrometry. Using the sensitive miRAMM, MGUS was estimated to be present in 887 of 17,367 persons from the Olmsted County cohort, translating into a prevalence of 5.1% among persons 50 years of age and older. Another study, using the MALDI-TOF mass spectrometry in a convenience cohort identified a higher prevalence of MGUS, but a significant proportion of the patients identified as having MGUS only demonstrated very small amount of IgM protein, likely a reactive phenomenon rather than true MGUS [29]. The relatively low proportion of IgM isotype among SPEP-IFE detected MGUS and even lower in symptomatic myeloma and amyloidosis supports this hypothesis.

**Fig. 4:**
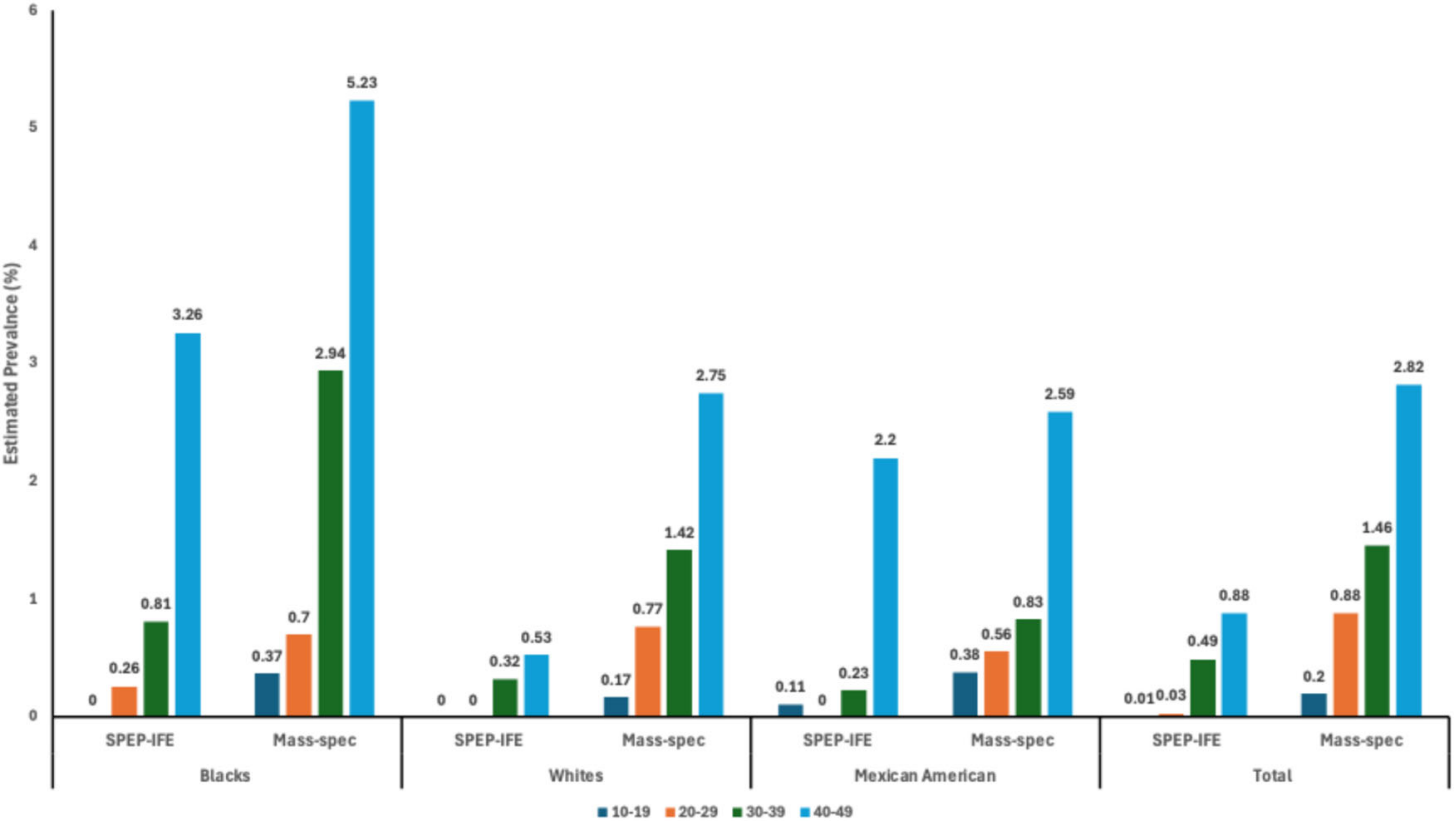
Prevalence of MGUS among 10–49-year-olds in NHANES III detected by SPEP+ immunofixation alone or with mass spectrometry.

The sensitivity of the methods used in our current study is reflected in the number of individuals with MGUS identified in the second and third decades of life. In our initial NHANES study using less sensitive electrophoretic methods, we identified MGUS in only two persons in the 10–19 age-group (both Mexican American), and in three persons in the 20–29-year age-group (all of whom were black) [2]. In contrast, we were able to identify 14 individuals with MGUS in the 10–19 and 28 individuals in the 20–29 age groups, respectively, translating into a 0.2% and 0.88% prevalence in the two age groups (Fig. 4), reflecting the higher sensitivity of the mass spectrometry assay. Identification of an early onset of MGUS has significant implications for identification of potential etiologies, especially related to environment exposures, as well as screening approaches and potential early interception efforts in MGUS.

The study also sheds light on potential explanation for the earlier onset by almost a decade of multiple myeloma among Black people, an observation that has been seen consistently in all epidemiological studies. The results here suggest that the earlier onset of myeloma is likely a reflection of the significantly earlier age of onset of MGUS. This is also supported by other studies that show that there is no impact of race on the risk of progression from MGUS to MM. While this finding was suggested by our earlier study using electrophoresis, there were too few patients in the earlier age groups to come to a definitive conclusion. In the current study we have been able to clearly demonstrate that among persons less than 50 years of age, MGUS is significantly more prevalent in Black people starting at age 30 compared to other racial groups.

As with prior studies, we again show a steady age-related increase in prevalence of MGUS across all races and both genders. This aligns well with the 4.4% prevalence observed in the Olmsted County population over 50 years of age, using the same technology, though that population is mostly Caucasian.

Based on prior studies suggesting an etiologic relationship between obesity and socioeconomic status [12, 36–39], we examined the potential association within this cohort, but we were not able to identify any such relationship. Lack of observed association does not necessarily rule out these relationships as they may very well be risk factors driving an accelerated progression of MGUS and may be operative at a later age. There was a suggestion of regional differences in this cohort based on the initial estimates using electrophoresis, but we did not observe that in the current analysis using the more sensitive assay.

In conclusion, this is the first study to define the timing of onset of MGUS using a highly sensitive mass spectrometry technique demonstrating an early onset of the clonal disorder as early as the second decade of life, conforming prior observations based on epidemiologic data and modeling. The observation of striking higher prevalence of MGUS in Black people starting at age 30 provides compelling explanation for the higher incidence of myeloma and related disorders in Black people that is apparent at age 40 onwards. Our data provides definitive information on the prevalence of MGUS by age, gender, and race in a younger population that has not been well studied in the past and is derived from a large population-based cohort enriched for minority populations. The fact that we can identify MGUS in the second decade of life should direct our investigations into the first decade of life for possible etiologies. The true prevalence using a sensitive test also provides the basis for exploring screening approaches for monoclonal gammopathies, which is not currently supported in the general population. However, the confirmation of racial differences can form the basis of studies exploring the role of screening for high-risk populations.

## Supplementary information

Suplementary Tables

## Data Availability

Data is publicly available at the NHANES website (https://wwwn.cdc.gov/nchs/nhanes/).
