# Supplementary material for: Prevalence of monoclonal gammopathy of undetermined significance (MGUS) using a sensitive mass spectrometry assay in young individuals 10–49 years of age: a population-based study from the National Health and Nutritional Examination Survey
Source: Blood Cancer J. 2026 May 25;16(1):100. doi: 10.1038/s41408-026-01518-7 (PMC13291244; doi:10.1038/s41408-026-01518-7)
Supplement: Supplementary file 1 — Suplementary Tables [file 41408_2026_1518_MOESM1_ESM.docx]

**SUPPLEMENTARY MATERIAL**

**Supplementary Table 1: Demographics by race/ethnicity and MGUS status^a^**

|  |  | **Race/ethnicity** | | | | **MGUS** | |
| --- | --- | --- | --- | --- | --- | --- | --- |
|  | **Total (N=12378)** | **Non-Hispanic White (N=3598)** | **Non-Hispanic Black (N=4075)** | **Mexican American (N=4147)** | **Other (N=558)** | **Negative/Indeterminate (N=12201)** | **Positive (N=177)** |
| **Age at entry** |  |  |  |  |  |  |  |
| N | 12378 | 3598 | 4075 | 4147 | 558 | 12201 | 177 |
| Mean (SD) | 27.8 (11.16) | 29.5 (11.26) | 27.4 (11.11) | 26.9 (10.93) | 27.0 (11.39) | 27.7 (11.13) | 36.2 (9.79) |
| Median | 27.0 | 30.0 | 27.0 | 26.0 | 26.0 | 27.0 | 38.0 |
| Range | 10.00, 49.00 | 10.00, 49.00 | 10.00, 49.00 | 10.00, 49.00 | 10.00, 49.00 | 10.00, 49.00 | 10.00, 49.00 |
| **Age Group**, n (%) |  |  |  |  |  |  |  |
| 10-19 | 3590 (29.0%) | 895 (24.9%) | 1265 (31.0%) | 1245 (30.0%) | 185 (33.2%) | 3576 (29.3%) | 14 (7.9%) |
| 20-29 | 3254 (26.3%) | 846 (23.5%) | 1024 (25.1%) | 1243 (30.0%) | 141 (25.3%) | 3226 (26.4%) | 28 (15.8%) |
| 30-39 | 3102 (25.1%) | 988 (27.5%) | 1039 (25.5%) | 947 (22.8%) | 128 (22.9%) | 3049 (25.0%) | 53 (29.9%) |
| 40-49 | 2432 (19.6%) | 869 (24.2%) | 747 (18.3%) | 712 (17.2%) | 104 (18.6%) | 2350 (19.3%) | 82 (46.3%) |
| **Sex**, n (%) |  |  |  |  |  |  |  |
| Male | 5712 (46.1%) | 1620 (45.0%) | 1815 (44.5%) | 2033 (49.0%) | 244 (43.7%) | 5635 (46.2%) | 77 (43.5%) |
| Female | 6666 (53.9%) | 1978 (55.0%) | 2260 (55.5%) | 2114 (51.0%) | 314 (56.3%) | 6566 (53.8%) | 100 (56.5%) |
| **Race/ethnicity**, n (%) |  |  |  |  |  |  |  |
| Non-Hispanic White | 3598 (29.1%) |  |  |  |  | 3547 (29.1%) | 51 (28.8%) |
| Non-Hispanic Black | 4075 (32.9%) |  |  |  |  | 3995 (32.7%) | 80 (45.2%) |
| Mexican American | 4147 (33.5%) |  |  |  |  | 4105 (33.6%) | 42 (23.7%) |
| Other | 558 (4.5%) |  |  |  |  | 554 (4.5%) | 4 (2.3%) |
| **Body mass index** |  |  |  |  |  |  |  |
| N | 12329 | 3591 | 4061 | 4122 | 555 | 12152 | 177 |
| Mean (SD) | 25.6 (6.20) | 24.8 (5.81) | 26.1 (6.89) | 25.8 (5.78) | 24.4 (5.59) | 25.6 (6.18) | 27.4 (6.90) |
| Median | 24.6 | 23.8 | 24.8 | 25.1 | 23.6 | 24.5 | 25.8 |
| Range | 11.20, 79.60 | 11.20, 67.30 | 13.50, 79.60 | 13.50, 59.70 | 14.50, 51.60 | 11.20, 79.60 | 14.90, 67.30 |
| **BMI class**, n (%) |  |  |  |  |  |  |  |
| Normal/underweight | 9859 (80.0%) | 3015 (84.0%) | 3103 (76.4%) | 3272 (79.4%) | 469 (84.5%) | 9730 (80.1%) | 129 (72.9%) |
| Obese | 2470 (20.0%) | 576 (16.0%) | 958 (23.6%) | 850 (20.6%) | 86 (15.5%) | 2422 (19.9%) | 48 (27.1%) |
| Missing | 49 | 7 | 14 | 25 | 3 | 49 | 0 |
| **Smoking (missing excluded)**, n (%) |  |  |  |  |  |  |  |
| Never | 2309 (92.6%) | 530 (86.9%) | 844 (95.5%) | 808 (93.1%) | 127 (96.9%) | 2302 (92.7%) | 7 (77.8%) |
| Former | 24 (1.0%) | 13 (2.1%) | 1 (0.1%) | 9 (1.0%) | 1 (0.8%) | 24 (1.0%) | 0 (0.0%) |
| Current | 160 (6.4%) | 67 (11.0%) | 39 (4.4%) | 51 (5.9%) | 3 (2.3%) | 158 (6.4%) | 2 (22.2%) |
| Missing | 9885 | 2988 | 3191 | 3279 | 427 | 9717 | 168 |
| **Poverty status**, n (%) |  |  |  |  |  |  |  |
| Below poverty | 8015 (70.6%) | 3096 (89.9%) | 2399 (64.3%) | 2189 (59.6%) | 331 (65.2%) | 7895 (70.5%) | 120 (74.1%) |
| Not below poverty | 3341 (29.4%) | 346 (10.1%) | 1333 (35.7%) | 1485 (40.4%) | 177 (34.8%) | 3299 (29.5%) | 42 (25.9%) |
| Missing | 1022 | 156 | 343 | 473 | 50 | 1007 | 15 |
| **Census region**, n (%) |  |  |  |  |  |  |  |
| South/West | 8624 (69.7%) | 2014 (56.0%) | 2642 (64.8%) | 3652 (88.1%) | 316 (56.6%) | 8506 (69.7%) | 118 (66.7%) |
| Northeast/Midwest | 3754 (30.3%) | 1584 (44.0%) | 1433 (35.2%) | 495 (11.9%) | 242 (43.4%) | 3695 (30.3%) | 59 (33.3%) |

^a^Unweighted summary

**Supplementary Table 2: Age, gender, and race-specific prevalence of MGUS (%)**

| Age group, years | Gender | Number with MGUS | Blacks (80)^a^  % (95% CI) | Whites (51)^a^  % (95% CI) | Mexican American (42)^a^  % (95% CI) | Total (177)^b^  % (95% CI) |
| --- | --- | --- | --- | --- | --- | --- |
| 10-19 | Male | 6 | 0.34 (0.00, 0.86) | 0.18 (0.00, 0.54) | 0.36 (0.00, 0.78) | 0.19 (0.00, 0.45) |
|  | Female | 8 | 0.39 (0.00, 0.89) | 0.16 (0.14, 0.19) | 0.39 (0.00, 0.91) | 0.20 (0.00, 0.42) |
|  | Total | 14 | 0.37 (0.01, 0.72) | 0.17 (0.00, 0.36) | 0.38 (0.00, 0.75) | 0.20 (0.02, 0.37) |
|  |  |  |  |  |  |  |
| 20-29 | Male | 16 | 1.17 (0.01, 2.32) | 0.86 (0.44, 1.28) | 0.64 (0.00, 1.33) | 0.99 (0.37, 1.61) |
|  | Female | 12 | 0.31 (0.00, 0.62) | 0.68 (0.00, 1.42) | 0.47 (0.06, 0.87) | 0.78 (0.09, 1.47) |
|  | Total | 28 | 0.70 (0.13, 1.27) | 0.77 (0.27, 1.27) | 0.56 (0.12, 1.01) | 0.88 (0.43, 1.34) |
|  |  |  |  |  |  |  |
| 30-39 | Male | 24 | 3.39 (1.62, 5.16) | 1.71 (0.12, 3.30) | 0.74 (0.00, 1.58) | 1.69 (0.48, 2.89) |
|  | Female | 29 | 2.58 (1.08, 4.07) | 1.14 (0.40, 1.88) | 0.94 (0.18, 1.70) | 1.23 (0.62, 1.85) |
|  | Total | 53 | 2.94 (1.89, 3.99) | 1.42 (0.54, 2.31) | 0.83 (0.20, 1.47) | 1.46 (0.78, 2.13) |
|  |  |  |  |  |  |  |
| 40-49 | Male | 31 | 4.04 (1.52, 6.56) | 1.38 (0.45, 2.30) | 3.38 (1.84, 4.93) | 1.69 (0.91, 2.48) |
|  | Female | 51 | 6.22 (3.83, 8.60) | 4.14 (2.11, 6.18) | 1.77 (0.53, 3.00) | 3.90 (2.34, 5.46) |
|  | Total | 82 | 5.23 (3.30, 7.16) | 2.75 (1.59, 3.92) | 2.59 (1.36, 3.83) | 2.82 (1.90, 3.74) |

Abbreviation: CI, confidence interval.

^a^Number in parentheses indicates number of people with MGUS in that race/ethnicity group.

^b^Includes ‘Other’ race/ethnicity group.

**Supplementary Table 3. Prevalence of MGUS (%) by race and poverty status**

| **Race** | **Poverty Status** | **Number with MGUS^b^** | **Prevalence of MGUS (%) (95% CI)** |
| --- | --- | --- | --- |
| Total^a^ | Below poverty | 120 | 1.22 (0.84, 1.60) |
|  | Not below poverty | 42 | 1.51 (0.34, 2.69) |
|  |  |  |  |
| Whites | Below poverty | 41 | 1.14 (0.68, 1.60) |
|  | Not below poverty | 7 | 2.16 (0.00, 4.86) |
|  |  |  |  |
| Blacks | Below poverty | 52 | 2.21 (1.55, 2.86) |
|  | Not below poverty | 22 | 1.89 (0.97, 2.80) |
|  |  |  |  |
| Mexican-American | Below poverty | 24 | 1.01 (0.60, 1.41) |
|  | Not below poverty | 13 | 0.69 (0.34, 1.04) |

Abbreviation: CI, confidence interval; NA, not applicable.

^a^Includes ‘Other’ race/ethnicity group.

^b^Because of missing poverty status data, there are 162/177 individuals with MGUS in this table
